# Translation, Cross‐Cultural Adaptation, and Validation of the Short Version of the Patterns of Activity Measure‐Pain in Brazilians With Chronic Pain

**DOI:** 10.1002/pri.70347

**Published:** 2026-09-16

**Authors:** Letícia Padilha Mendes, Ailka Barros Barbosa, Devyd Weyder do Nascimento Freitas, Ana Maria Araújo Viana, Ana Clara Oliveira Santana, Flávio de Oliveira Pires, Almir Vieira Dibai‐Filho

**Affiliations:** ^1^ Postgraduate Program in Physical Education Universidade Federal do Maranhão São Luís Maranhão Brazil; ^2^ Department of Physical Therapy Centro Universitário Florence São Luís Maranhão Brazil; ^3^ Department of Physical Education Universidade Federal de Maranhão São Luís Maranhão Brazil

**Keywords:** activity patterns, chronic pain, cross‐cultural, validation studies, questionnaires

## Abstract

**Objective:**

To translate, cross‐culturally adapt, and validate the short version of the Patterns of Activity Measure‐Pain (POAM‐P) in Brazilian individuals with chronic pain.

**Methods:**

This was a questionnaire validation study. Brazilian participants reporting pain for more than 3 months were included. The measurement properties assessed were structural validity, construct validity, reliability, internal consistency, and agreement.

**Results:**

The study included 125 individuals with chronic pain for the analysis of structural and construct validity. The internal structure of the reduced version of the POAM‐P, comprising 3 domains and 15 items, was considered adequate (comparative fit index = 0.98; Tucker‐Lewis index = 0.97; root mean square error of approximation = 0.07; standardized root mean square residual = 0.06; and chi‐square/degrees of freedom = 1.55). Correlations ranging from 0.30 to 0.50 were observed between the avoidance and pacing domains and kinesiophobia, catastrophizing, and disability. The overdoing domain showed no significant correlations with external measures, associating negatively only with the avoidance and pacing domains. Internal consistency was assessed in the total sample (*n* = 125) using composite reliability (CR), with values ranging from 0.76 to 0.93 across the three POAM‐P domains. Test–retest reliability was assessed in a subsample of 33 individuals and proved satisfactory, with intraclass correlation coefficients ≥ 0.79. The test–retest interval was planned to range from 7 to 14 days, with an observed mean of 8 days (7–13). Agreement, assessed via Bland‐Altman plots in the same subsample (*n* = 33), was considered acceptable. No ceiling or floor effects were identified.

**Conclusion:**

The Brazilian short version of the POAM‐P demonstrated adequate structural validity, internal consistency, and test–retest reliability. Nevertheless, further studies with larger retest samples are warranted to confirm the stability of these findings. Regarding construct validity, the avoidance and pacing domains showed correlations with external measures as expected, whereas the overdoing domain was correlated only with the other domains of the instrument.

## 1 Introduction

It is common for individuals living with pain to exhibit characteristic activity patterns, as described in the fear‐avoidance model. Within this framework, catastrophic thoughts and avoidance behaviors toward daily activities are commonly observed (Zale and Ditre 2015). In addition to avoidance, another important activity pattern is overdoing, characterized by persistence in performing daily activities despite pain and often disregarding symptoms (Serrano‐Ibáñez et al. 2020). This behavior may contribute to increased functional disability, particularly when associated with excessive persistence and maladaptive responses to pain (H. P. J. Kindermans et al. 2011).

Notably, persistence and avoidance are not necessarily mutually exclusive, as individuals may alternate between periods of excessive activity and activity avoidance following pain exacerbation. Although avoidance and overdoing are generally considered as maladaptive activity patterns, pacing has been proposed as a third pattern. Pacing involves regulating or adjusting activity levels according to pain intensity and physical capacity, with the aim of maintaining functionality and preventing symptom exacerbation; however, it can also be considered maladaptive (Cane et al. 2013; H. Kindermans et al. 2009).

To the best of our knowledge, an important tool used to assess aspects related to chronic pain and activity is the Patterns of Activity Measure‐Pain (POAM‐P). It is a self‐report questionnaire developed in 2013, comprising 30 items, each with four alternatives, scored from 0 to 4 points. The POAM‐P consists of three domains: avoidance, overdoing, and pacing. Its original English version showed adequate measurement properties for the questionnaire's target population (Cane et al. 2013). Subsequently, a short version with 15 items and 3 domains was developed, showing adequate measurement properties and correlations with the long form of the questionnaire (Cane and Mazmanian 2020).

The long version of the POAM‐P has already been translated and validated into Dutch (H. Kindermans et al. 2009), French (Benaim et al. 2017), Turkish (Suygun and Celenay 2022), and Japanese (Enomoto et al. 2022). In addition, the Spanish version was only translated and had its reliability was verified (Esteve et al. 2016). However, the short version of the POAM‐P only exists in its original English version (Cane and Mazmanian 2020), despite instruments with a smaller number of items having several positive characteristics, such as: reduced completion time, reduction in the number of completion errors, reduction in the number of unanswered items and optimizing clinical and scientific applicability (do Nascimento Freitas et al. 2024; Fidelis‐de‐Paula‐Gomes et al. 2023).

Considering the above and the aspects mentioned, in Brazil, to date, there is no validated and cross‐culturally adapted version of the short version of the POAM‐P to measure these three outcomes simultaneously, which prevents its use in this population, the creation of intervention strategies, and the comparison of results with the original study. Therefore, the objective of this study was to translate, cross‐culturally adapt, and validate the short version of the POAM‐P in Brazilians with chronic pain.

## 2 Methods

This was a questionnaire validation study conducted according to the Guidelines for the Process of Cross‐cultural Adaptation of Self‐Report Measures (Beaton et al. 2000) and the Consensus‐based Standards for the Selection of Health Measurement Instruments (COSMIN) (Prinsen et al. 2018).

Authorization to perform the translation of the short version of the POAM‐P into Brazilian Portuguese was granted via email by one of the questionnaire's authors (Dr. Douglas Cane). The study was conducted in three phases: translation and cross‐cultural adaptation of the POAM‐P, testing of the pre‐final version of the translated POAM‐P into Brazilian Portuguese, and validation of the final version of the POAM‐P cross‐culturally adapted into Brazilian Portuguese.

### 2.1 Translation and Cross‐Cultural Adaptation

The procedures for the translation and cross‐cultural adaptation of the short version of the POAM‐P into Brazilian Portuguese followed the criteria of Beaton et al. (2000) and were carried out in stages, as described below.

1. Translation: The translation process was performed by two independent translators (one physiotherapist with 17 years of experience in the field and one English teacher with 26 years of translation experience but no technical knowledge of health‐related subjects). Both were native Brazilian Portuguese speakers fluent in English. They independently translated the original short version of the POAM‐P into Brazilian Portuguese.
2. Synthesis of the translations: After discussions and revisions, the two translators, under the observation of one of the lead researchers, synthesized the two independently translated versions of the questionnaire and produced a single, consensual short version of the POAM‐P.
3. Back‐translation: Two independent translators without technical knowledge of health‐related subjects, both native English speakers fluent in Portuguese, performed the back‐translation. They translated the short Portuguese version of the POAM‐P back into English, without any prior knowledge of the original questionnaire.
4. Expert committee analysis: A committee of 4 rehabilitation specialists, together with the 4 translators involved in the project, was formed. This committee analyzed and reviewed all translated and back‐translated versions to correct possible discrepancies, thus achieving a consensual pre‐final short version of the POAM‐P agreed upon by all committee members.
5. Pre‐final version testing: The pre‐final short version of the POAM‐P was administered to 30 individuals with pain in any body part for more than 3 months (chronic pain) and who were native Brazilian Portuguese speakers. The individuals read and completed the questionnaire, and at the end, they reported their understanding of the pre‐final version of the POAM‐P by marking a selection box containing the answers “yes” and “no” for each questionnaire item. To be considered as having an adequate level of comprehension, the items had to be understood by more than 80% of the participants.
6. Final version testing: To verify the measurement properties of the final Brazilian Portuguese short version of the POAM‐P.

### 2.2 Sample Size and Eligibility Criteria

For translation, our sample size was based on international recommendations. Initially, it consisted of 30 individuals for testing the pre‐final version (Beaton et al. 2000). To verify the validity of the instrument, the final version of the POAM‐P was applied to 125 individuals, following the COSMIN recommendation that the minimum sample size should be seven times the number of items in the questionnaire (15 items × 7 = 105 individuals). For the test–retest reliability analysis, a target sample size of 50 participants was established. All 125 participants included in the main study were invited to participate in the retest (Terwee et al. 2011).

Individuals of both sexes, aged ≥ 18 years, with self‐reported chronic pain lasting more than 3 months in any region of the body and able to read and understand Brazilian Portuguese were included. Individuals diagnosed with infectious diseases, neurological disorders, cancer, or conditions that prevented the completion of the questionnaire, as well as individuals with a Numeric Pain Rating Scale (NPRS) score of 0, were excluded from the study.

Since all questionnaire items were mandatory in both the online and in‐person formats, and all responses were reviewed by the researchers for completeness and consistency, it was not possible for there to be any missing items.

### 2.3 Recruitment and Data Collection

Data collection was conducted online using the Google Forms platform (Mountain View, CA, United States) and at a physiotherapy school clinic in the city of São Luís (Maranhão, northeastern Brazil). Participants were recruited through verbal dissemination, poster advertisements, and social media. The recruitment flyers included a QR code that directed potential participants to a single online link containing all study materials. The same procedure was followed for participants recruited in person at the physiotherapy school clinic.

Participants recruited at the physiotherapy school clinic were patients receiving care at the clinic and members of the general community. Those recruited through social media, verbal dissemination, and poster advertisements were members of the general community. Participants were not exclusively students of the physiotherapy school.

All study information was collected through a single questionnaire, with no separate assessment or screening forms. Personal characteristics, including age, sex, weight, height, and body mass index (BMI), were collected through self‐report together with the study questionnaires, using the same link for online participants and the same questionnaire for participants assessed in person. Thus, personal characteristics and questionnaire responses were collected as part of the same data collection procedure, and no personal characteristic information was collected separately from the questionnaires.

All 125 participants included in the study were invited to participate in the test–retest assessment. The intended sample size for the test–retest assessment was 50 participants. Upon completing the initial questionnaire, participants were informed that they would be invited to complete the second stage of the assessment (retest) again within the planned 7‐ to 14‐day interval (Terwee et al. 2011). No reminder questionnaires were sent to participants to increase response rates. All participants provided informed consent before participation. The research procedures were approved by the Institutional Research Ethics Committee (opinion number 5.984.526).

### 2.4 Assessments

We collected sociodemographic information as part of the online questionnaires, including (sex, age, marital status, and education), anthropometric measures (body mass, height, and body mass index), and clinical variables (pain location and duration, presence of treatment, and systemic, metabolic, rheumatologic, and cardiovascular comorbidities). These data were used to screen participants for eligibility based on the predefined inclusion and exclusion criteria.

### 2.5 Patterns of Activity Measure‐Pain (POAM‐P)

The short version of the POAM‐P consists of 15 items divided into 3 domains: five items for the avoidance domain, five items for the overdoing domain, and five items for the pacing domain. For each item, possible responses range from “never” (equivalent to 0) to “all the time” (equivalent to 4) to best describe how an individual usually performs their daily activities in the face of pain. The scores for the three domains are given by the sum: avoidance, items 2, 5, 7, 11, and 14; overdoing, items 1, 3, 6, 9, and 12; and pacing, items 4, 8, 10, 13, and 15. The score for each domain ranged from 0 to 20 points. The higher the score, the greater the activity pattern the individual presents when performing their daily activities (Cane and Mazmanian 2020). The Brazilian version of the POAM‐P can be obtained free of charge at questionariosbrasil.blogspot.com.

### 2.6 Other Questionnaires

For construct validation, correlations were performed between the short version of the POAM‐P and the following instruments validated for Brazilian Portuguese: NPRS, Tampa Scale for Kinesiophobia (TSK), Pain Catastrophizing Scale (PCS), and Roland Morris Disability Questionnaire for general pain (RMDQ‐g).

### 2.7 Numeric Pain Rating Scale (NPRS)

The NPRS is a simple and easily measurable scale that consists of a sequence of numbers from 0 to 10, where the value 0 represents “no pain” and the number 10 represents “the worst pain imaginable.” Thus, individuals rated their pain based on these parameters. The individual identifies the maximum pain intensity at the time (Ferreira‐Valente et al. 2011).

### 2.8 Tampa Scale of Kinesiophobia (TSK)

This scale consists of a self‐administered questionnaire comprising 17 items related to fear of movement. The scores range from 1 to 4 points, where the response 1 means “strongly disagree,” 2 “disagree,” 3 “agree,” and 4 “strongly agree.” To obtain the final score, the scores for items 4, 8, 12, and 16 must be reversed. The final score can range from a minimum of 17 to a maximum of 68 points, where a higher score indicates a greater degree of kinesiophobia presented by the individual (Siqueira et al. 2007).

### 2.9 Pain Catastrophizing Scale (PCS)

The PCS is a scale that assesses pain‐related catastrophizing. It consists of 13 items and is divided into three domains: helplessness, magnification, and rumination. The scores range from zero to four points, where the response 0 means “not at all,” 1 “to a slight degree,” 2 “to a moderate degree,” 3 “to a great degree,” and 4 “all the time.” The scores for the three domains are given by the sum of helplessness, items 1–5 and 12; magnification, items 6, 7, and 13; and rumination, items 8–11. The score for the rumination domain ranges from 0 to 16, magnification from 0 to 12, and helplessness from 0 to 24 points. The higher the score, the greater the degree of catastrophizing (Sehn et al. 2012).

### 2.10 Roland Morris Disability Questionnaire for General Pain (RMDQ‐g)

The RMDQ‐g was validated and adapted for the Brazilian population with general pain by Sardá Júnior et al. (2010). It is an instrument that assesses functional disability related to general chronic pain, consisting of 24 items describing daily activities, where the participant marks the item that describes their current situation. For each marked statement, a score of 1 is quantified, so that the total score ranges from 0 to 24. The higher the total score, the greater the disability (Sardá Júnior et al. 2010).

### 2.11 Global Perceived Effect Scale (GPES)

This scale was applied only at the retest to the 33 individuals to identify clinical stability, with an interval of 7–14 days between assessments. The GPES consists of an 11‐point scale, where −5 indicates “much worse,” 0 indicates “no change,” and +5 indicates “completely recovered,” with higher scores indicating greater recovery from the condition (Costa et al. 2008). Clinical stability was considered as a variation of less than or equal to 2 points.

### 2.12 Statistical Analysis

Sociodemographic data were described as mean and standard deviation (quantitative data) or as absolute number and percentage (qualitative data). The following tests were performed to verify the questionnaire's measurement properties.

Structural validity was analyzed via confirmatory factor analysis (CFA) using R Studio software (Boston, MA, United States), with the utilization of the *lavaan* and *semPlot* packages. The analysis was performed using a polychoric covariance matrix and the robust diagonally weighted least squares (RDWLS) extraction method, given that the POAM‐P scores are of an ordinal categorical nature. The following indices were considered to verify the model fit: comparative fit index (CFI) and Tucker‐Lewis index (TLI) ≥ 0.90; root mean square error of approximation (RMSEA) and standardized root mean square residual (SRMR) ≤ 0.08; and chi‐square/degrees of freedom (DF) ≤ 3 (Brown 2006; Schermelleh‐Engel et al. 2003).

To determine construct validity through correlations between instruments, the normality of the data was initially verified using the Kolmogorov‐Smirnov test. Subsequently, Spearman's correlation coefficient (rho) was used to determine the magnitude of the correlation between the POAM‐P domains and the other instruments in the study. The interpretation of the correlation magnitudes followed these criteria: instruments measuring similar constructs should be ≥ 0.50; instruments measuring related but different constructs should be between 0.30 and 0.50, and correlations with instruments measuring unrelated constructs should be < 0.30 (Prinsen et al. 2018).

We hypothesized a priori that the POAM‐P domains would show correlation magnitudes ranging from 0.30 to 0.50 with the instruments used in this study. Specifically, the avoidance domain was expected to show positive and significant correlations, the overdoing domain was expected to show negative correlations with the outcomes assessed, and the pacing domain was expected to show positive and significant correlations.

Internal consistency was assessed using composite reliability (CR) and value ≥ 0.70 was considered adequate (Raykov 1997). We used the online calculator from The Statistical Mind, available at: https://thestatisticalmind.com/comp‐reliabily/. Test–retest reliability was assessed with an interval of 7–14 days between evaluations using the intraclass correlation coefficient (ICC_2,1_). The interpretation of the ICC_2,1_ value was based on the study by Fleiss (1999): values below 0.40 were considered low reliability; values between 0.40 and 0.75 were moderate; values between 0.75 and 0.90 were substantial; and finally, values greater than 0.90 were considered excellent reliability. Two measures were used to assess the measurement error: standard error of measurement (SEM) and minimal detectable change (MDC). The mathematical formulas for calculating SEM and MDC are described in the study by Bassi et al. (2018). The agreement between the results obtained from the short version of the POAM‐P at test and retest was assessed using Bland‐Altman plots.

The ceiling and floor effects were evaluated in this study. These occur when a proportion of study participants (more than 15%) achieve the minimum or maximum total score of a questionnaire domain, indicating limitations in the instrument's ability to discriminate between different levels of the construct (Terwee et al. 2011).

The processing for descriptive analysis, construct validity, reliability, agreement, and ceiling and floor effects was performed using the Statistical Package for the Social Sciences (SPSS), version 17.0 (Chicago, IL, United States). A significance level of 5% was adopted for the analyses.

## 3 Results

### 3.1 Translation and Cross‐Cultural Adaptation

During the translation phase, no adaptations of terms were necessary, and the pre‐final version was established by consensus by the expert committee. The pre‐final version of the POAM‐P was then applied to 30 individuals with chronic pain, mostly women (52.9%), single (70.6%), with incomplete higher education (38.2%), and who reported predominantly lower back pain. None of the questionnaire items were misinterpreted by more than 20% of the individuals. Therefore, the pre‐final version of the POAM‐P was established as the final Brazilian Portuguese version of the POAM‐P. None of the 30 individuals who participated in the pre‐final version testing were included among the individuals who participated in the validation of the final version.

### 3.2 Sample Characterization

The sample consisted of 129 individuals. However, there was a sample loss of 4 participants because they had a score of 0 on NPRS, indicating the absence of pain at the time of assessment and therefore not meeting the study eligibility criteria. That is, 125 individuals were included, the majority of whom were women, with a mean age of 38 years, single, overweight, having completed higher education, suffering from chronic low back pain, with a mean pain score of 5.73 and a mean pain symptom chronicity duration of 43 months. It should be noted that 55.2% of the sample was undergoing treatment, which could be physiotherapy, since some of the participants were recruited from a physiotherapy teaching clinic. Other information is described in Tables 1 and 2.

**TABLE 1:** Descriptive analysis of quantitative personal characteristics for the full sample (*n* = 125) and subsample (*n* = 33).

Characteristics	Mean (standard deviation)	Mean (standard deviation)
	Full sample (*n* = 125)	Subsample (*n* = 33)
Age (years)	38.08 (14.46)	35.00 (13.14)
Body mass (kg)	73.38 (15.10)	72.12 (15.26)
Height (m)	1.65 (0.13)	1.65 (0.10)
Body mass index (kg/m^2^)	26.55 (4.88)	26.06 (4.09)
Pain duration (months)	43.51 (64.59)	36.06 (34.09)
NPRS (score, 0–10)	5.73 (2.23)	5.94 (2.24)
POAM‐P (score, 0–20)		
Avoidance	7.30 (4.53)	7.12 (4.46)
Overdoing	12.00 (4.40)	12.06 (4.38)
Pacing	8.00 (5.53)	8.79 (5.05)
TSK (score, 17–68)	38.49 (7.57)	38.85 (6.49)
PCS		
Helplessness (score, 0–24)	6.62 (4.75)	7.67 (4.29)
Magnification (score, 0–12)	4.60 (2.96)	5.24 (3.09)
Rumination (score, 0–16)	6.63 (4.27)	6.88 (4.21)
RMDQ‐g (score, 0–24)	7.38 (5.98)	6.33 (5.87)

Abbreviations: NPRS, Numeric Pain Rating Scale; PCS, Pain Catastrophizing Scale; POAM‐P, Patterns of Activity Measure‐Pain; RMDQ‐g, Roland‐Morris Disability Questionnaire for general chronic pain; TSK, Tampa Scale for Kinesiophobia.

**TABLE 2:** Descriptive analysis of categorical personal and clinical characteristics for the full sample (*n* = 125) and subsample (*n* = 33).

Characteristics	Number (%)	Number (%)
	Full sample (*n* = 125)	Subsample (*n* = 33)
Gender		
Female	77 (61.6)	20 (60.6)
Male	48 (38.4)	13 (39.4)
Marital status		
Single	76 (60.8)	24 (72.7)
Married	37 (29.6)	6 (18.2)
Divorced	9 (7.20)	2 (6.1)
Widowed	2 (1.60)	1 (3.0)
Common‐law marriage	1 (0.8)	0 (0)
Education level		
Complete elementary education	3 (2.4)	0 (0)
Incomplete elementary education	1 (0.8)	0 (0)
Complete high school education	17 (13.6)	5 (15.2)
Incomplete high school education	2 (1.6)	0 (0)
Complete higher education	32 (25.6)	5 (15.2)
Incomplete higher education	30 (24.0)	10 (30.3)
Complete postgraduate education	31 (24.8)	9 (27.3)
Incomplete postgraduate education	9 (7.2)	4 (12.1)
Pain location		
Low back	48 (38.4)	13 (39.4)
Knee	19 (15.2)	4 (12.1)
Cervical	14 (11.2)	3 (9.1)
Shoulder	13 (10.4)	2 (6.1)
Ankle	4 (3.2)	2 (6.1)
Thoracic	2 (1.6)	0 (0)
Wrist	2 (1.6)	2 (6.1)
Upper back	1 (0.8)	0 (0)
Others	22 (17.2)	7 (21.1)
Pain treatment		
Yes	69 (55.2)	18 (54.5)
No	56 (44.8)	15 (45.5)
Systemic disease		
Yes	20 (16.0)	6 (18.2)
No	105 (84.0)	27 (81.8)
Metabolic disease		
Yes	7 (5.6)	2 (6.1)
No	118 (94.4)	31 (93.9)
Rheumatologic disease		
Yes	29 (23.2)	8 (24.2)
No	96 (76.8)	25 (75.8)
Cardiovascular disease		
Yes	3 (2.4)	0 (0)
No	122 (97.6)	33 (100.0)

### 3.3 Structural Validity

**FIGURE 1:**
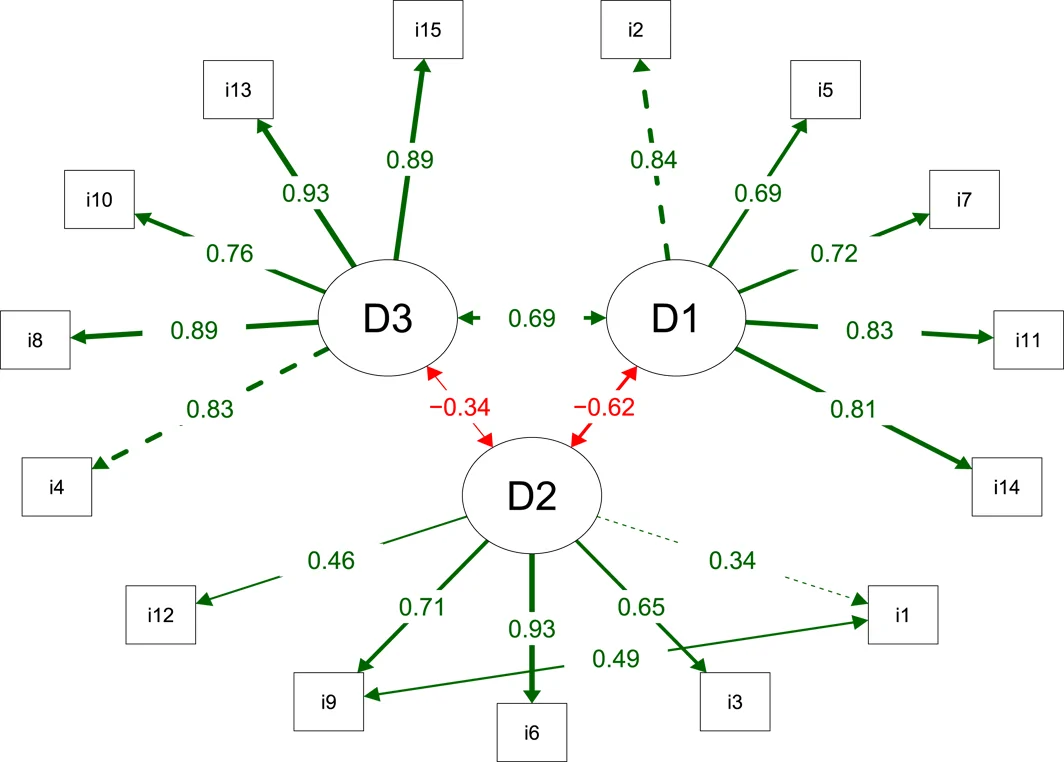
Path diagram with factor loadings of the short version of the Patterns of Activity Measure‐Pain (POAM‐P). D1: avoidance domain; D2: overdoing domain; D3: pacing domain. The dotted line represents the first item of each domain, and its thickness is proportional to the factor loading.

Based on the internal structure presented by the authors who developed the short version of the POAM‐P (i.e., 3 domains and 15 items), we performed a CFA and observed adequate fit indices: CFI = 0.98, TLI = 0.97, RMSEA (90% confidence interval) = 0.07 (0.04–0.09), SRMR = 0.06, chi‐square/DF = 1.55. Regarding the factor loadings, as shown in Figure 1, only item 1, corresponding to the overdoing domain, showed a factor loading of 0.34, below the recommended value of 0.40. Nevertheless, considering the satisfactory fit indices, the original structure of the short version of the POAM‐P was maintained.

### 3.4 Construct Validity

As shown in Table 3, all correlations in the avoidance domain exhibited a positive direction, with 83.33% displaying a magnitude between 0.30 and 0.50, as hypothesized. For the pacing domain, all correlations also showed a positive direction, and 50% presented a magnitude between 0.30 and 0.50, as hypothesized. Regarding the overdoing domain, all correlations displayed a negative direction; however, none reached the magnitude expected in our prior hypothesis.

**TABLE 3:** Correlation between the domain scores of the short version of the Patterns of Activity Measure‐Pain (POAM‐P) and the other questionnaires in the present study (*n* = 125).

Variables	Domain 1		Domain 2		Domain 3	
	Rho	*p*	Rho	*p*	Rho	*p*
TSK	0.425	< 0.001	−0.175	0.051	0.403	< 0.001
PCS						
Helplessness	0.304	0.001	−0.020	0.822	0.259	0.004
Magnification	0.310	< 0.001	−0.016	0.857	0.309	< 0.001
Rumination	0.329	< 0.001	−0.044	0.627	0.282	0.001
RMDQ‐g	0.381	< 0.001	−0.154	0.087	0.345	< 0.001
NPRS	0.205	0.022	−0.085	0.349	0.082	0.366

*Note:* Domain 1: avoidance; Domain 2: overdoing; Domain 3: pacing; rho: Spearman's correlation coefficient.

Abbreviations: NPRS, Numeric Pain Rating Scale; PCS, Pain Catastrophizing Scale; RMDQ‐g, Roland‐Morris Disability Questionnaire; TSK, Tampa Scale for Kinesiophobia.

Considering only the correlations among the POAM‐P domains, we observed a negative correlation of the overdoing domain with both the avoidance domain (rho = −0.62, *p* = 0.004) and the pacing domain (rho = −0.34, *p* = 0.020). A positive correlation was also found between the avoidance and pacing domains (rho = 0.69, *p* < 0.001), as illustrated in Figure 1.

### 3.5 Reliability and Internal Consistency

Adequate test–retest reliability was observed, with ICC_2,1_ values ≥ 0.79 for all domains of the short version of the POAM‐P. Although a 7‐ to 14‐day interval was planned between administrations, the actual test–retest interval had a mean of 8 days, ranging from 7 to 13 days, with the mean interval falling within the planned. Of the 125 participants invited to complete the test–retest assessment, 33 completed the second administration, corresponding to a retention rate of 26.4%. Clinical stability of the 33 participants was confirmed by the GPES (variation ≤ 2 points). Internal consistency was also adequate (*n* = 125), with composite reliability (CR) from 0.76 to 0.93 across the three POAM‐P domains. Further information is described in Table 4.

**TABLE 4:** Test–retest reliability (*n* = 33) and internal consistency (*n* = 125) of the short version of the Patterns of Activity Measure‐Pain (POAM‐P).

Domains	Test	Retest	ICC	95% CI	SEM (score)	MDC (score)	Composite reliability
Domain 1	7.12 (4.46)	9.45 (5.61)	0.79	0.61 a 0.89	2.31	6.40	0.88
Domain 2	12.06 (4.38)	12.06 (4.53)	0.88	0.77 a 0.93	1.54	4.28	0.76
Domain 3	8.79 (5.05)	8.27 (4.28)	0.86	0.75 a 0.93	1.75	4.84	0.93

*Note:* Domain 1: avoidance; Domain 2: overdoing; Domain 3: pacing.

Abbreviations: CI, confidence interval; ICC, intraclass correlation coefficient; MDC, minimal detectable change; SEM, standard error of measurement.

### 3.6 Agreement

**FIGURE 2:**
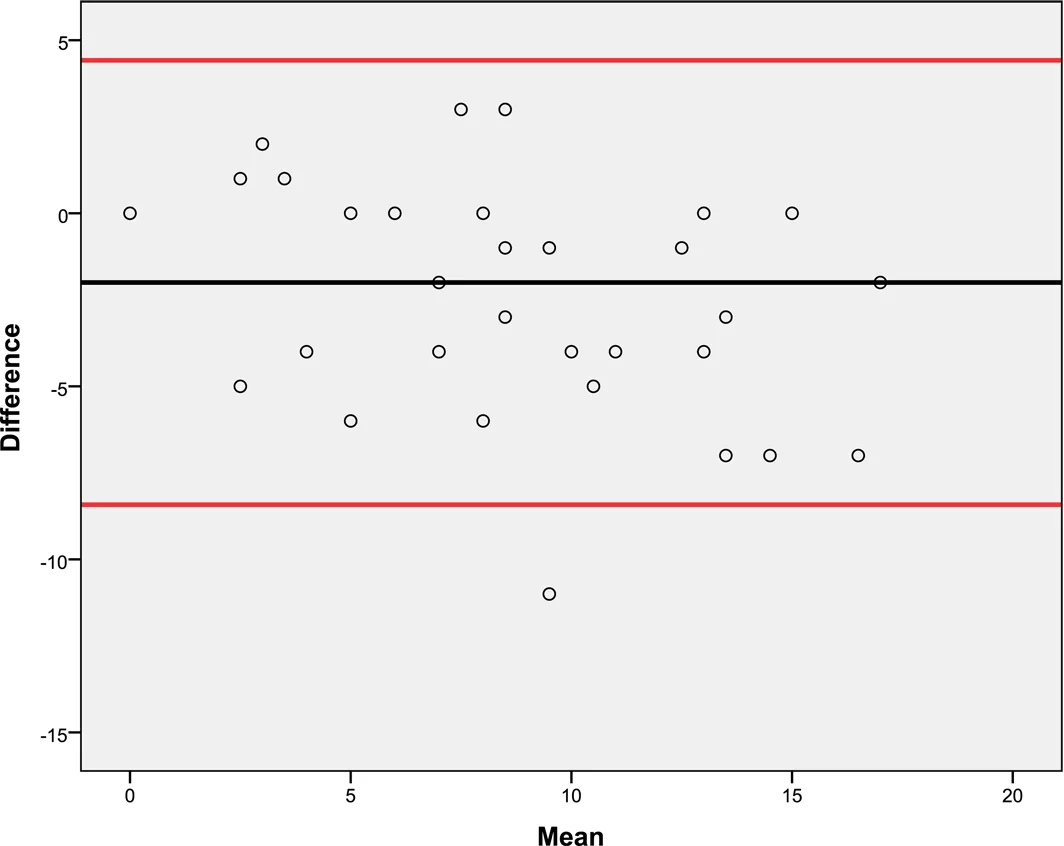
Agreement plot between test and retest for domain 1 (avoidance) of the short version of the Patterns of Activity Measure‐Pain (POAM‐P).

**FIGURE 3:**
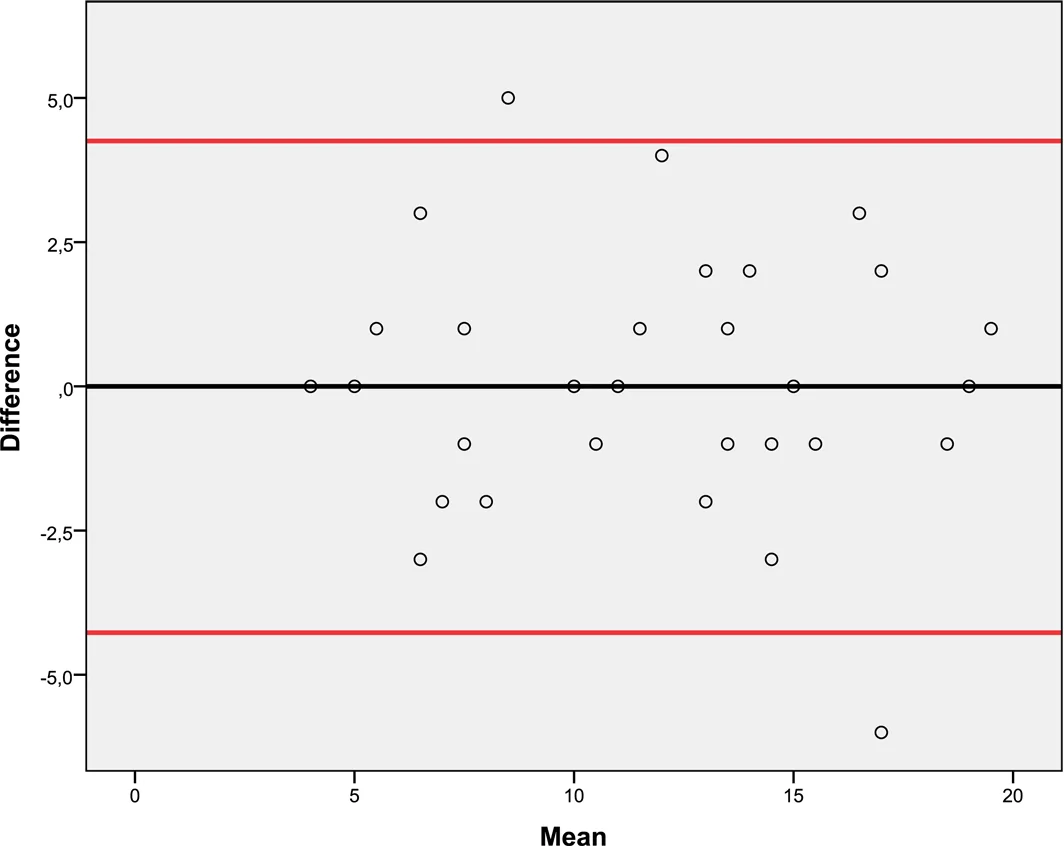
Agreement plot between test and retest for domain 2 (overdoing) of the short version of the Patterns of Activity Measure‐Pain (POAM‐P).

**FIGURE 4:**
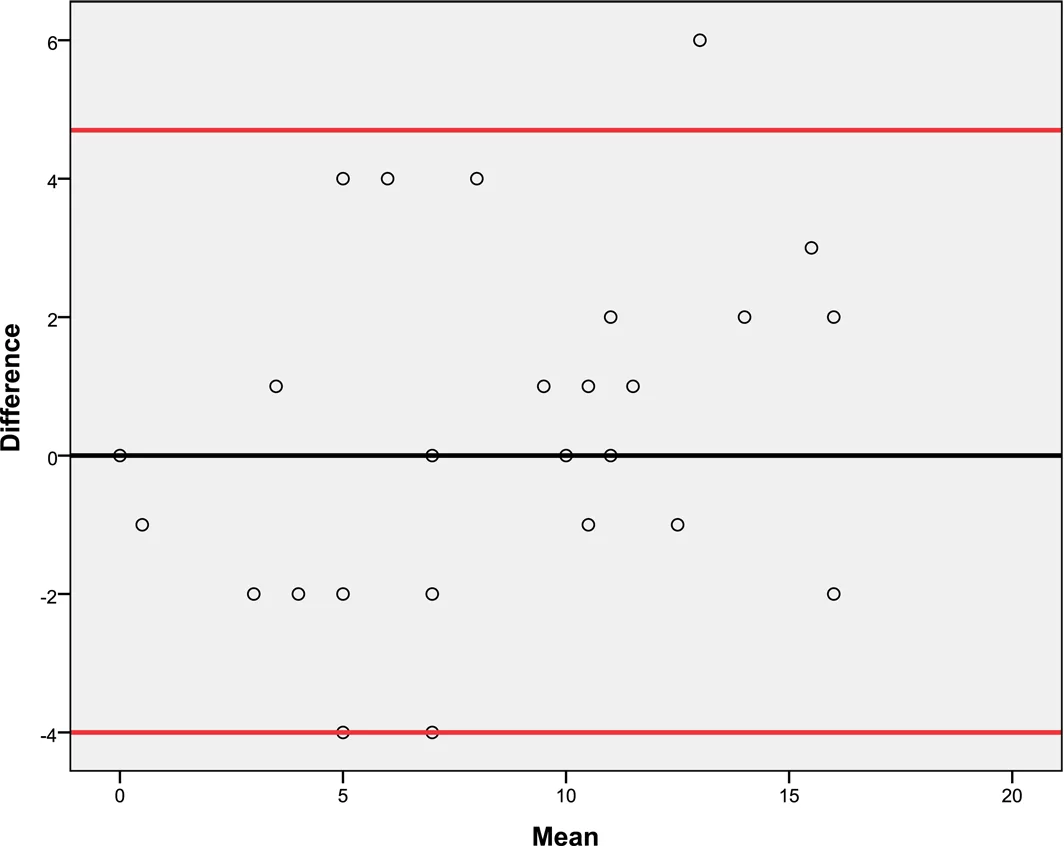
Agreement plot between test and retest for domain 3 (pacing) of the short version of the Patterns of Activity Measure‐Pain (POAM‐P).

The Bland–Altman plots were performed based on the test–retest reliability results (*n* = 33), considering the scores of each domain of the short version of the POAM‐P. Figures 2, 3, 4 present the test–retest agreement for each domain. The mean difference between measurements was −2.00 for avoidance, with 95% limits of agreement ranging from −8.42 to 4.42. For overdoing, the mean difference was 0.00, with limits of agreement ranging from −4.27 to 4.27, while for pacing, the mean difference was 0.00, with limits ranging from −4.70 to 4.70.

Thus, overdoing and pacing showed no average systematic bias, whereas avoidance showed a small negative mean difference. Overall, the Bland–Altman analysis indicated reasonable test–retest agreement although the wider limits of agreement for avoidance indicate greater individual variability between assessments.

### 3.7 Ceiling and Floor Effects

Regarding the avoidance domain, 12 (9.6%) individuals achieved the minimum score of 0 and no one achieved the maximum score of 20 points. In the overdoing domain, no one achieved the minimum score and 4 (3.2%) individuals achieved the maximum score. Regarding the pacing domain, 14 (11.2%) individuals achieved the minimum score and 3 (2.4%) individuals achieved the maximum score. Thus, no floor or ceiling effects were observed.

## 4 Discussion

The present study aimed to perform the translation, cross‐cultural adaptation, and validation of the short version of the POAM‐P in Brazilians with chronic pain, which were undertaken. Furthermore, the instrument exhibits a valid internal structure with 3 domains and 15 items, adequate reliability, and construct validity that was partially confirmed, while still presenting good applicability in clinical practice and research. No ceiling or floor effects were found.

Most participants in the present study reported pain in the lumbar region. This finding is similar to that observed in validation studies of the instrument, in which low back pain was also predominant, such as in the Turkish version, which reported a prevalence of 60.7% (Suygun and Celenay 2022). The present study included a younger sample (mean age = 38.08 years) compared with previous validations of the long (47.8–49.2 years) and short (49.5–52.2 years) versions of the POAM‐P (Cane et al. 2013; Cane and Mazmanian 2020). This characteristic may have contributed to higher overdoing scores, as younger individuals may show greater persistence in maintaining occupational and social activities, pushing themselves to their limits regardless of pain intensity or fear of symptom exacerbation.

To the best of our knowledge, the English short version (Cane and Mazmanian 2020) used classical test theory for item‐total correlation in item selection. This differs from our study, which employed factor analysis, but we identified adequate fit indices that confirm the structure of the original short version of the POAM‐P.

To date, we have not identified a questionnaire or scale with constructs identical to the POAM‐P. Therefore, we examined correlations with instruments measuring related constructs and observed correlation magnitudes ranging from 0.30 to 0.50, partially confirming the study hypothesis. The avoidance domain correlated with pain intensity, catastrophizing, kinesiophobia, and disability, suggesting that higher levels of avoidance are associated with greater fear of movement, catastrophizing, disability, and pain intensity, thereby corroborating our hypothesis.

The pacing domain correlated positively with magnification (catastrophizing domain), kinesiophobia, and disability (rho between 0.30 and 0.50), such that higher pacing scores were associated with higher levels of these measures. In contrast, the association between pacing and numerical pain intensity was not statistically significant (*p* = 0.366). These correlation magnitudes confirmed 50% of our a priori hypotheses. This hypothesis was based on previous findings suggesting that pacing may be associated with greater disability and pain‐related difficulties (Benaim et al. 2017; H. Kindermans et al. 2009). Similarly, some dimensions of pacing were associated with worse symptoms in a validation study using the 12‐item Activity Pacing Questionnaire (APQ‐12), supporting the multidimensional nature of pacing (Antcliff et al. 2025). However, the APQ‐12 has not yet been validated in Brazilian Portuguese.

In contrast, the overdoing domain did not show significant correlations with the external instruments, including disability, similar to the Dutch sample, suggesting that individuals with this activity pattern may maintain their activity levels despite pain (H. Kindermans et al. 2009). The correlation between the POAM‐P overdoing subscale and kinesiophobia was negative (rho = −0.175, *p* = 0.051), aligning with the hypothesized direction. However, it reached neither statistical significance nor the expected correlation magnitude. Consequently, these findings do not support the hypothesized relationship and should be interpreted with caution.

Within the POAM‐P, overdoing was negatively correlated with both avoidance (rho = −0.62, *p* = 0.004) and pacing (rho = −0.34, *p* = 0.020), indicating that higher overdoing scores were associated with lower scores on avoidance and pacing. In contrast, pacing was positively correlated with avoidance (rho = 0.69, *p* < 0.001), such that higher pacing scores were associated with higher avoidance scores. These findings suggest distinct relationships among the activity patterns assessed by the POAM‐P, with overdoing showing an inverse relationship with both avoidance and pacing, whereas pacing and avoidance were positively related.

The English short version of the POAM‐P was not correlated with measures of catastrophizing and kinesiophobia, but showed a correlation with a magnitude between 0.30 and 0.50 only for the avoidance domain with the Pain Disability Index. The other observed correlations ranged from 0.03 to 0.29 (Cane and Mazmanian 2020). Thus, the present study showed correlations of greater magnitude than those reported in the original validation. This difference may be partly explained by differences in sample characteristics and outcome measures between the studies. In particular, the present study included a younger sample and used measures of kinesiophobia and pain catastrophizing, whereas the original study assessed perceived pain control and depression in addition to pain intensity and disability (Cane and Mazmanian 2020).

Furthermore, a slightly similar correlation magnitude was observed in the English long version with the TSK for both the avoidance (0.42) and overdoing (−0.26) domains (Cane et al. 2013). In the French long version, the avoidance and pacing domains of the POAM‐P were correlated with the TSK and the PCS, with values ranging between 0.134 and 0.508. Regarding the overdoing domain, the correlation values were −0.276 (with the PCS total score) and −0.289 (with the TSK) (Benaim et al. 2017).

Regarding test–retest reliability, the ICC_2,1_ values found in the present study ranged from 0.79 to 0.88, indicating that it is an instrument consistent over time. The short version of the POAM‐P describes as a limitation the non‐assessment of this measurement property (Cane and Mazmanian 2020). In the long version, ICC_2,1_ scores for test–retest reliability were not analyzed (Cane et al. 2013).

Regarding internal consistency, we identified adequate CR values among the domains (≥ 0.76), as in the original short version (Cronbach's alpha ≥ 0.89) (Cane and Mazmanian 2020) and the original long version (Cronbach's alpha ≥ 0.86) (Cane et al. 2013).

Our study has limitations that should be considered. Firstly, there are no other studies that have validated the short version of this instrument for other languages, a fact that limited the discussion and comparison with more studies. Secondly, data collection was conducted in a hybrid manner (in‐person and online), and the diagnosis was based on self‐report. In addition, data collection in a virtual environment requires access to the internet and electronic devices, which may have hindered the participation of Brazilians in situations of social vulnerability in the study. There were few responses in the retest, a factor that compromised the sample size for reliability.

The overdoing domain did not correlate with the external measures. Concurrent validity was also not examined, as overdoing and activity pacing were not compared with other specific measures of these constructs. Another limitation was that the responsiveness of the Brazilian version of the POAM‐P was not assessed. Furthermore, although the sample size met the COSMIN recommendation, we acknowledge that the sample for the structural validity analysis was smaller than the 200 participants frequently recommended for CFA (Mokkink et al. 2024). In addition, the limited sample may restrict the generalizability of the findings.

In terms of clinical applicability, it is essential to consider that the overdoing domain may help identify individuals with a tendency toward work overload and difficulties in balancing professional and personal demands. Thus, even in the absence of significant correlations with the external instruments used, this domain may capture specific aspects of functioning that are clinically relevant.

## 5 Conclusion

The Brazilian short version of the POAM‐P demonstrated adequate structural validity, internal consistency, and test–retest reliability. Nevertheless, further studies with larger retest samples are warranted to confirm the stability of these findings. Construct validity was partially supported for the Avoidance and Pacing domains. However, the Overdoing domain requires further investigation using external measures more closely aligned with activity persistence and overactivity.

## Funding

The study received funding from Fundação de Amparo à Pesquisa e ao Desenvolvimento Científico e Tecnológico do Maranhão (FAPEMA, code BM‐09859/24) and Coordenação de Aperfeiçoamento de Pessoal de Nível Superior (CAPES, code 001).

## Conflicts of Interest

The authors declare no conflicts of interest.

## Supporting information


Supporting Information S1

## Data Availability

The data that support the findings of this study are available on request from the corresponding author. The data are not publicly available due to privacy or ethical restrictions.
