# Supplementary material for: Translation, Cross‐Cultural Adaptation, and Validation of the Short Version of the Patterns of Activity Measure‐Pain in Brazilians With Chronic Pain
Source: Physiother Res Int. 2026 Sep 16;31(4):e70347. doi: 10.1002/pri.70347 (PMC13583028; doi:10.1002/pri.70347)
Supplement: Supplementary file 1 [file PRI-31-e70347-s001.docx]

**Short-Form of the Patterns of Activity Measure-Pain (SF-POAM-P)**

Nome: ________________________________________________________ Data: _______________

As pessoas que sentem dor utilizam maneiras diferentes de realizar suas atividades diárias. Pense em como você costuma fazê-las. Para cada uma das afirmações abaixo, circule um número entre 0 e 4 que melhor descreve como você costuma realizar essas atividades.

|  |  | **Nunca** | **Às vezes** | **O tempo todo** |
| --- | --- | --- | --- | --- |
|  |  |  |  |  |
| 1. | Eu continuo fazendo o que estou fazendo até que minha dor seja tão forte que eu tenha que parar. | 0 1 2 3 4 | | |
| 2. | Eu evito atividades que eu sei que irão piorar minha dor. | 0 1 2 3 4 | | |
| 3. | Quando eu começo uma atividade, eu continuo até terminar ela. | 0 1 2 3 4 | | |
| 4. | Quando faço uma atividade, eu a divido em pequenas partes e faço uma parte de cada vez. | 0 1 2 3 4 | | |
| 5. | Eu só faço aquelas atividades que sei que não irão piorar a minha dor. | 0 1 2 3 4 | | |
| 6. | Eu simplesmente ignoro minha dor e continuo fazendo a atividade até o máximo que aguentar. | 0 1 2 3 4 | | |
| 7. | Eu não começo uma atividade se sei que irá piorar a minha dor. | 0 1 2 3 4 | | |
| 8. | Em vez de realizar uma atividade de uma só vez, faço um pouco de cada vez. | 0 1 2 3 4 | | |
| 9. | Eu continuo fazendo a atividade até não suportar mais a dor. | 0 1 2 3 4 | | |
| 10. | Eu me lembro de fazer pausas quando estou fazendo uma atividade. | 0 1 2 3 4 | | |
| 11. | Se eu sei que algo vai fazer a minha dor piorar, não faço mais. | 0 1 2 3 4 | | |
| 12. | Quando eu realizo uma atividade, faço-a por completo de uma só vez. | 0 1 2 3 4 | | |
| 13. | Em vez de fazer a atividade por completo, eu a divido em pequenas partes e faço uma parte de cada vez. | 0 1 2 3 4 | | |
| 14. | Eu reduzi as minhas atividades por não fazer aquelas que pioram a minha dor. | 0 1 2 3 4 | | |
| 15. | Quando eu faço uma atividade, faço-a por um tempo, paro e depois volto a ela. | 0 1 2 3 4 | | |
